# The use of bedside echocardiography for measuring cardiac index and systemic vascular resistance in pediatric patients with septic shock

**DOI:** 10.5935/0103-507X.20180067

**Published:** 2018

**Authors:** Faten A. Abdalaziz, Hebat Allah Fadel Algebaly, Reem Ibrahim Ismail, Seham Awad El-Sherbini, Ahmed Behairy

**Affiliations:** 1 Department of Pediatrics, Faculty of Medicine, Cairo University - Cairo, Egypt.

**Keywords:** Echocardiography, Hemodynamic, Septic shock, Child

## Abstract

**Objective:**

Follow-up of cardiac index and systemic vascular resistance index by bedside echocardiography until resuscitation.

**Methods:**

A set of hemodynamic parameters was obtained, including cardiac output, stroke volume, cardiac index, systemic vascular resistance index, velocity time integral, myocardial performance index, capillary refill time, and heart rate at 0 hours after fluid boluses before the start of inotropes, and followed up after 6 hours and 24 hours.

**Results:**

Included were 45 patients with community-acquired septic shock. Septic foci were gastroenteritis (24%), intestinal perforation requiring emergency surgery (24%), pneumonia (20%), central nervous system infection (22%) and soft tissue infection (8%). Klebsiella and Enterobacter were the most frequent isolates. We estimated the factors affecting the cardiac index: high central venous pressure at zero time (r = 0.33, p = 0.024) and persistently high heart rate at hour 6 (r = 0.33, p = 0.03). The systemic vascular resistance index was high in most patients at 0 and 24 hours and at the time of resuscitation and inversely affected the cardiac index as well as affecting the velocity time integral (r = -0.416, -0.61, 0.55 and -0.295). Prolonged capillary refill time was a clinical predictor of the low velocity time integral at 24 hours (r = -0.4). The mortality was 27%. Lower systemic vascular resistance index and higher cardiac output were observed in nonsurviving patients.

**Conclusion:**

There was a persistently high systemic vascular resistance index in cold shock patients that influenced the stroke volume index, cardiac index, and velocity time integral. The use of echocardiograms for hemodynamic measurements is important in pediatric septic shock patients to adjust dilators, and vasopressor doses and achieve resuscitation targets in a timely manner.

## INTRODUCTION

Early aggressive fluid resuscitation, antibiotics, and vasoactive therapies are recommended by the American College of Critical Care Medicine as clinical practice parameters for hemodynamic support of septic shock in children and neonates; they also recommend echocardiography to guide fluid and vasoactive regimens as well as to rule out pericardial effusion.^(1^^),(^^2)^

The hemodynamic monitoring of shock in the intensive care unit (ICU) is of great value for diagnosis of septic shock as one of the differential diagnoses of circulatory shock, evaluating the hemodynamic condition involving the detection of therapeutic conflicts, and directing the treatment regimen.^(3)^

Septic shock in children is classically differentiated into either cold shock (low cardiac index, often high systemic vascular resistance index - SVRI), and warm shock (high cardiac index, low SVRI).^(4)^

In applying any hemodynamic monitoring technique, cardiac index is considered the reference standard parameter for targeting organ perfusion and oxygen delivery in shock.^(5)^

Cardiac output is classically evaluated by insertion of a pulmonary artery catheter (Swan-Ganz) using the thermodilution technique.^(6)^ There is an increased interest in replacing this invasive method with noninvasive or minimally invasive technologies to measure cardiac index, including pulse contour analysis, lithium dye dilution, electrical bioimpedance, and transesophageal and transthoracic echocardiography with pulsed or continuous wave Doppler ultrasound.^(7)^ A focused ultrasound cardiac examination to evaluate cardiac index, SVRI, and other echo parameters have been discussed in recent studies as a possible method used by emergency room (ER) physicians for the management of critically ill patients.^(8^^),(^^9)^ It was found that increased cardiac index was associated with better renal function and better renal outcome in patients with acute renal injury.^(10)^

Systemic vascular resistance index was found to be a powerful predictor of mortality in children with septic shock because low values of SVRI denote endothelial layer injuries that are one of the important pathophysiologies of sepsis.^(11)^

Left ventricular outflow tract-velocity time integral (LVOT-VTI) (used to calculate stroke volume) is thought to be a better indicator of LV systolic function than stroke volume, without the confounding factor of LVOT-area measurements, which are difficult to obtain in ICU patients.^(12)^

We aimed to study the role of bedside echocardiography as a noninvasive tool in assessing the hemodynamic changes in critically ill patients with septic shock with the hope of helping manage such patients.

## METHODS

This prospective observational cohort study was conducted between June 2014 and July 2015, in an academic pediatric intensive care unit (PICU) of Cairo University Specialized Hospital with a 14-bed capacity and approximately 300 yearly admissions. The study was approved by the institutional review board at our institution and was deemed to pose minimal risk to the subjects. Before study enrollment, a PICU physician was trained to measure the echocardiographic parameters using the ultrasound system echocardiographic equipment (GE, Logic P3). Training of the PICU physician included a total of 48 hours of hands-on instruction by a certified cardiac sonographer to obtain the LVOT-VTI and the Doppler mitral inflow signal to be able to calculate cardiac index, SVRI, myocardial performance index (Tei index) and fractional shortening (FS).

Patients with strong clinical suspicion of septic shock at the time of admission to the PICU were included. Septic shock was diagnosed according to the American College of Critical Care Medicine Clinical Practice Parameters for Hemodynamic Support of Pediatric and Neonatal Septic Shock 2012 by clinical signs, including hypothermia or hyperthermia, altered mental status, and peripheral vasodilation (warm shock) or vasoconstriction with capillary refill time (CRT) greater than 2 seconds (cold shock) before hypotension occurs.^(13)^ All cases included were considered to be in a state of fluid refractory septic shock when they received at least 40mL/kg shock fluids (normal saline or Ringer's) and required inotropic/vasopressor support for hemodynamic resuscitation, whether ventilated or not.

Patients with congenital heart diseases, cardiomyopathy, and valvular disorders were excluded. After informed consent was obtained from the caregiver, transthoracic two-dimensional, M-mode, and Doppler echocardiography was performed on commercially available echocardiographic equipment (GE, Logic P3) using a 6S probe. Echocardiography was performed to measure the LVOT diameter measured in the long-axis parasternal view, and the time-velocity integral of the flow wave across the aortic valve (VTI) was obtained by pulsed wave Doppler. All ultrasound images obtained by the PICU physician and sonographer were stored and then verified by a cardiologist using a scale to rate the acceptability of the ultrasound measurements.

Left ventricular outflow tract-velocity time integral is a Doppler-derived measure of the distance traveled by midstream blood through the left ventricular outflow tract in a single cardiac cycle, i.e., stroke distance. Left ventricular outflow tract diameter was obtained by measuring the distance from inner edge to inner edge, where the right aortic valve coronary cusp meets the interventricular septum to where the noncoronary cusp meets the anterior mitral valve leaflet, in a line parallel to the aortic annulus.^(4)^ Stroke volume was calculated by the equation: π × VTI × (LVOT diameter/2)^2^. Stroke volume index (SVI) = stroke volume/body surface area. Cardiac output = stroke volume × heart rate. Cardiac index = cardiac output/ body surface area. M-mode echocardiography was done to measure left ventricular end systolic diameter (LVESD), left ventricular end diastolic diameter (LVEDD), FS is measured by the equation: LVEDD - LVESD/LVEDD × 100. Myocardial performance index (Tei Index) was calculated by the equation: the sum of isovolumic contraction time and isovolumic relaxation time (total systolic time- ejection time) divided by the ejection time obtained by imaging the Doppler mitral inflow signal. Myocardial performance index - MPI (Tei index) = Total systolic time- ejection time/ejection time. Systemic vascular resistance was calculated by the equation: 80 (mean blood pressure-central venous pressure)/cardiac index.^(14)^

All cases were subjected to full clinical and echocardiographic examination at the time of PICU admission before inotropic support (Time 0), after 6 hours, after 24 hours and then at the time of stabilization (resuscitation point), which was identified by normalization of heart rate (resolved tachycardia), blood pressure (resolved hypotension if present), CRT (less than 2 seconds), good urine output (≥ 1mL/kg per hour), and regaining of consciousness with the start of vasoactive drug withdrawal.

Different vasoactive agents were used in management of the study cases with fluid refractory septic shock. These agents included adrenaline, noradrenaline, dobutamine, milrinone, and dopamine; overt vasodilator agents such as nitroglycerin were also used.

Inotropic score (IS) was calculated by the Wernowsky equation:

$$\begin{array}{c} \mathrm{Wernowsky}\;\mathrm{IS}=\;\mathrm{dopa}\min e\;\mathrm{dose}+\mathrm{dob}u\mathrm{ta}\min e\;\mathrm{dose}+\\ 100\times \mathrm{epinephire}\;{\mathrm{dose}}^{(15)} \end{array}$$

Vasoactive IS (VIS) was calculated by the following equation:

$$\begin{array}{c} \mathrm{VIS}=\mathrm{IS}+10\times \mathrm{milrinone}\;\mathrm{dose}\;+10,000\times \\ \mathrm{vasopres}\sin\;\mathrm{dose}+100\times \mathrm{norepinephrine}\;{\mathrm{dose}}^{(16)} \end{array}$$

### Statistical analysis

Data were analyzed using the Statistical Package of Social Science Software (SPSS) program, version 21.

Data were summarized using mean and standard deviation for quantitative variables and frequency and percentage for qualitative variables.

Comparison between groups was performed using independent sample t-tests or one-way ANOVA with post hoc Tukey's tests for quantitative variables and chi square or Fisher's exact tests for qualitative variables.

Paired t-tests were conducted to compare quantitative data.

Pearson or Spearman correlation coefficients were calculated to test the association between parametric and nonparametric variables, respectively.

P values < 0.05 were considered statistically significant, and < 0.01 were considered highly significant.

## RESULTS

In this study, 45 patients with fluid refractory septic shock were included. There were 26 (57.8%) males and 19 (42.2%) females. Their mean age was 20.1 ± 29.1 months (2 - 120 months) and mean body weight was 9.8 ± 6.2kg. Their mean CRT at time of admission was 5 ± 0.9 seconds, and mean heart rate at the time of admission was 153 ± 24bpm. The most common causes of sepsis were gastroenteritis and postoperative sepsis followed by CNS infections. The most common isolated organism in blood culture was *Klebsiella* followed by *Enterobacter*. No growth was detected in 66.7% of cases, as shown in table 1. Of the 45 cases, 33 (73%) patients improved and were discharged from the PICU, and 12 (27%) patients died.

**Table 1:** Clinical and laboratory characteristics of the study population

Variables	
Age (months)	20.1 ± 29.1
Weight (kg)	9.8 ± 6.2
Heart rate (bpm)	153 ± 24
CVP (mmHg)	4.6 ± 3.4
SBP (mmHg)	96 ± 17
CRT (seconds)	5.0 ± 0.9
Sex	
Males	26 (57.8)
Females	19 (42.2)
Septic focus	
Gastroenteritis	11 (24.4)
CNS infection	10 (22.2)
Lung infection	9 (20)
Skin infection	3 (6.7)
Postoperative	11 (24.4)
Blood stream	1 (2.3)
Type of organism	
No growth	30 (66.7)
*Klebsiella*	5 (11.2)
MRSA	1 (2.2)
*Enterobacter*	3 (6.7)
CONS	1 (2.2)
*Candida non-albicans*	2 (4.4)
*Acinetobacter*	2 (4.4)
*Escherichia coli*	1 (2.2)

CVP - central venous pressure; SBP - systolic blood pressure; CRT - capillary refill time; MRSA - Methicillin-resistant *Staphylococcus aureus*; CONS - Coagulase Negative *Staphylococci*. Results expressed as mean ± standard deviation or n (%).

Duration of resuscitation from septic shock was assessed. Thirty (66.7%) of 45 cases were resuscitated before 24 hours, and the median resuscitation time of all survivors was 34 hours.

Thirty-seven (82%) of 45 cases had echocardiographic criteria of cold septic shock with low normal or low cardiac index (≤ 3.3L/m/m^2^) and normal or high SVRI (≥ 1,600dyn-sec/cm^5^/m^2^), whereas 8 (18%) cases had echocardiographic criteria of warm septic shock (high cardiac index > 6L/m/m^2^) with low normal or low SVRI (≤ 800dyn-sec/cm^5^/m^2^).

**Figure 1:**
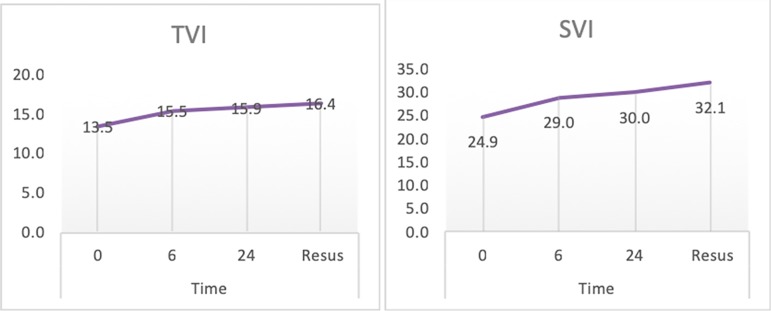
Line curve showing pattern of time velocity integral and stroke volume index measured at different time intervals. TVI - time velocity integral; SVI - stroke volume index.

Bedside echocardiography for measurement of hemodynamic indices over several time intervals was performed. Comparing these indices assessed at time 0 (T0) and time of stabilization (resuscitation point), we found that the cardiac index, SVI, VTI showed dynamic improvement over time (Table 2, Figure 1). We was found that FS remained constant with no statistical change, whereas MPI (Tei index) was significantly increased by the time of resuscitation of septic shock (0.3 ± 0.1 at admission versus 0.4 ± 0.1 by mean time of resuscitation, with p value 0.008), MPI showed a negative correlation with the central venous pressure (r = -0.3; p = 0.04)*.*

**Table 2:** The echocardiographic data assessed at time of admission, after 6 hours and at the time of resuscitation

Clinical/echocardiography data	Admission data	After 6 hours	After 24 hours	Resuscitation point	Data on admission versus time of resuscitationp value
Heart rate (beats/minute)	153 ± 24	154 ± 24	142.7 ± 25.2	138 ± 22	0.003
CRT (seconds)	5 ± 0.9	3.2 ± 1	2.3 ± 1.3	1.8 ± 1.1	< 0.001
TVI	13.5 ± 3.7	15.5 ± 3.6	15.9 ± 4.1	16.4 ± 3.9	< 0.001
SV (mL)	11.3 ± 7.1	13.1 ± .7	14.1 ± 8.7	14.6 ± 8.1	0.001
SVI (mL/beat)	24.9 ± 8.3	29 ± 7.9	30 ± 8.4	32.1 ± 8.1	0.001
CO (mL/minute)	1.653 ± 0.888	1.938 ± 0.919	1.908 ± 0.917	1.914 ± 0.832	0.06
CI (L/m/m2)	3.7 ± 1.2	4.4 ± 1.1	4.2 ± 1.1	4.4 ± 1.1	< 0.001
FS (%)	35.0 ± 8.4	36.2 ± 7.0	37.4 ± 6.8	37.4 ± 6.2	0.13
SVRI (dyn sec/cm^5^/m^2^)	1.514 ± 550	1.398 ± 535	1.487.9 ± 430.6	1.402 ± 400	0.218
Tei index	0.3 ± 0.1	0.4 ± 0.1	0.4 ± 0.1	0.4 ± 0.1	0.043

CRT - capillary refill time; TVI - time velocity integral; SV - stroke volume; SVI - stroke volume index; CO - cardiac output; CI - cardiac index; FS - fraction shortening; SVRI - systemic vascular resistance index; TEI index - myocardial performance index, (resuscitation): measures taken at the time of resuscitated septic shock. Results expressed as mean ± standard deviation.

**Figure 2:**
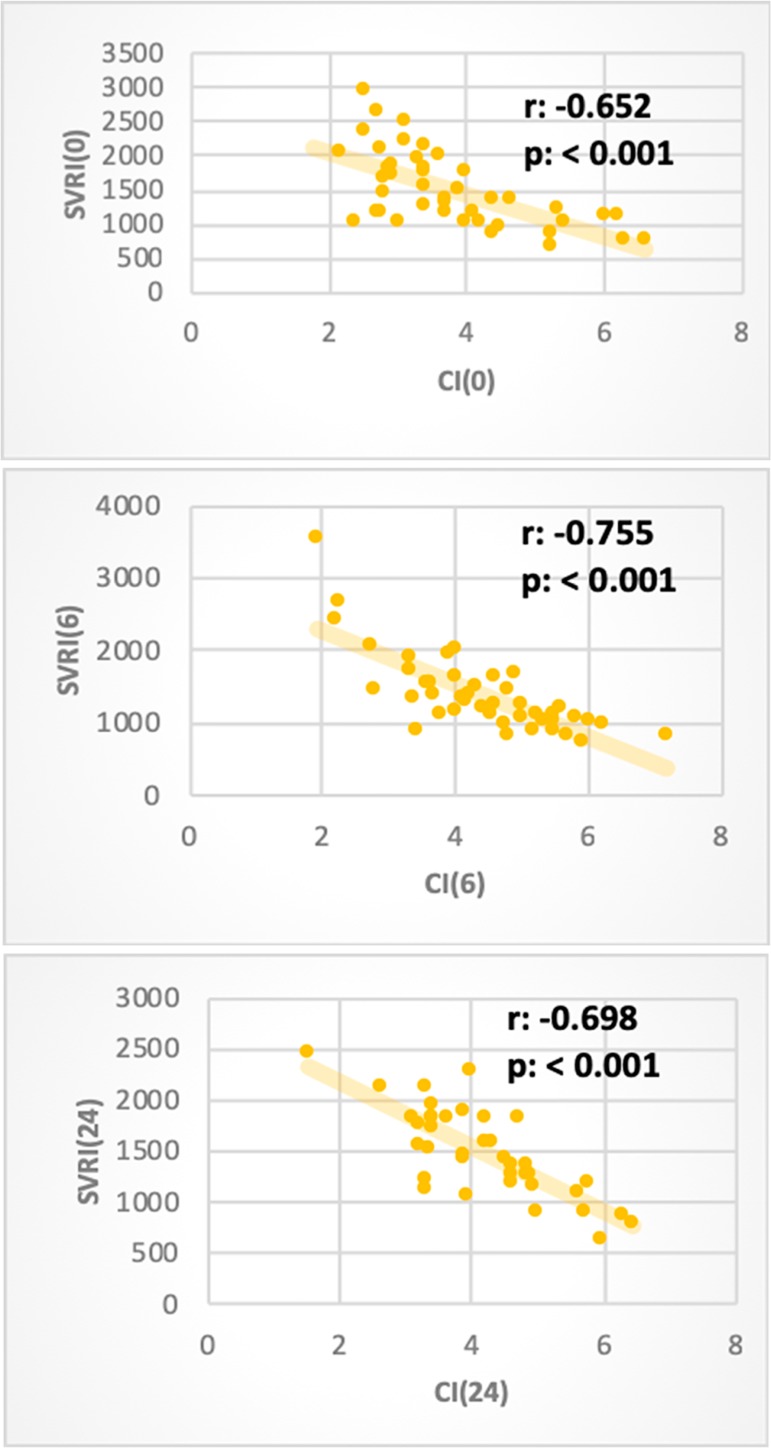
Showing the significant negative correlation between the cardiac index and systemic vascular resistance index at different time intervals. SVRI - systemic vascular resistance index; CI - cardiac index.

It was interesting to find that the initial SVRI was 1,514 ± 550 dyn-sec/cm^5^/m^2^. SVRI declined to 1,398 ± 535dyn-sec/cm^5^/m^2^ at 6 hours, and then had a rebound elevation at the mean time of resuscitation (34 hours) to 1,402 ± 400dyn-sec/cm^5^/m^2^ (Table 2). The SVRI was the only measured index that showed a second rise at the time point of resuscitation in cases with cold shock, and we intended to see its effect as afterload on the other hemodynamic parameters. We found that VTI, SVI, and cardiac index were negatively affected by the SVRI at various time intervals. The heart rate and the myocardial performance index were not affected by the SVRI (Table 3, Figure 2).

**Table 3:** Correlations between systemic vascular resistance index with other variables at different time intervals

	Systemic vascular resistance index			
	Admission	After 6 hours	After 24 hours	Resuscitation point
TVI				
r	-0.416	-0.617	-0.559	-0.295
p value	0.006	< 0.001	< 0.001	0.049
SVI				
r	-0.509	-0.736	-0.637	-0.533
p value	< 0.001	< 0.001	< 0.001	< 0.001
HR				
r	-0.124	-0.160	-0.050	-0.159
p value	0.427	0.294	0.765	0.298
CI				
r	-0.652	-0.755	-0.698	-0.717
p value	< 0.001	< 0.001	< 0.001	< 0.001
LVESD				
r	-0.227	-0.048	0.039	-0.202
p value	0.143	0.755	0.818	0.182
LVEDD				
r	-0.263	-0.049	-0.026	-0.245
p value	0.088	0.747	0.877	0.105
FS				
r	-0.029	-0.219	-0.165	-0.003
p value	0.854	0.149	0.323	0.986
CVP				
r	-0.364	-0.033	0.019	-0.166
p value	0.016	0.828	0.911	0.276
TEI				
r	-0.082	0.002	-0.144	0.117
p value	0.621	0.990	0.389	0.443
CRT				
r	-0.277	0.087	0.385	0.242
p value	0.072	0.569	0.017	0.109

TVI - time velocity integral; SVI - stroke volume index; HR - heart rate; CI - cardiac index; LVESD - left ventricular end systolic diameter; LVEDD - left ventricular end diastolic diameter; FS - fraction shortening; CVP - central venous pressure; TEI index - myocardial performance index; CRT - capillary refill time.

The cardiac index was found to have a linear positive correlation with SVI and VTI assessed at different time intervals and had a negative correlation with SVRI (Table 4).

**Table 4:** Correlations between cardiac index and other variables at different time intervals

	Cardiac index			
	Admission	After 6 hours	After 24 hours	Resuscitation point
LVOT				
r	0.050	0.118	0.175	0.257
p value	0.744	0.440	0.293	0.089
TVI				
r	0.775	0.613	0.526	0.371
p value	< 0.001	< 0.001	0.001	0.012
SVI				
r	0.858	0.842	0.719	0.776
p value	< 0.001	< 0.001	< 0.001	< 0.001
HR				
r	0.094	0.337	0.257	0.204
p value	0.539	0.023	0.118	0.178
LVESD				
r	0.136	0.082	-0.011	0.162
p value	0.374	0.591	0.947	0.289
LVEDD				
r	0.171	0.020	-0.009	0.150
p value	0.261	0.898	0.959	0.326
FS				
r	0.105	0.173	0.002	-0.062
p value	0.494	0.255	0.990	0.684
CVP				
r	0.341	0.011	-0.142	-0.003
p value	0.024	0.941	0.395	0.984
MBP				
r	0.133	0.154	0.042	0.104
p value	0.391	0.312	0.801	0.496
SVRI				
r	-0.652	-0.755	-0.698	-0.717
p value	< 0.001	< 0.001	< 0.001	< 0.001
TEI				
r	-0.030	0.057	-0.014	-0.028
p value	0.852	0.717	0.936	0.855
CRT				
r	0.078	-0.320	-0.262	-0.237
p value	0.613	0.032	0.112	0.117

LVOT - left ventricular outflow tract diameter; TVI - time velocity integral; SVI - stroke volume index; HR - heart rate; LVESD - left ventricular end systolic diameter; LVEDD - left ventricular end diastolic diameter; FS - fraction shortening; CVP - central venous pressure; MBP - mean blood pressure; SVRI - systemic vascular resistance index; TEI index - myocardial performance index; CRT - capillary refill time.

Velocity-time integral was negatively affected by the rising SVRI at different time intervals and with CRT at 24 hours (Table 5).

**Table 5:** Correlations between time velocity integral and other variables at different time intervals

	Time velocity integral of aortic outflow signal			
	Admission	After 6 hours	After 24 hours	Resuscitation point
SVRI				
r	-0.416	-0.617	-0.559	-0.295
p value	0.006	<0.001	<0.001	0.049
LVOT				
r	0.035	0.118	0.157	0.100
p value	0.819	0.442	0.345	0.512
LVESD				
r	0.116	0.202	0.240	0.400
p value	0.450	0.184	0.146	0.006
LVEDD				
r	0.221	0.261	0.293	0.370
p value	0.144	0.084	0.074	0.012
FS				
r	0.109	0.277	0.028	-0.142
p value	0.476	0.066	0.869	0.352
CVP				
r	0.278	0.091	-0.056	0.051
p value	0.068	0.552	0.737	0.742
TEI				
r	0.025	-0.117	-0.196	-0.213
p value	0.878	0.454	0.238	0.160
CRT				
r	0.116	-0.243	-0.393	-0.197
p value	0.448	0.107	0.015	0.195

SVRI - systemic vascular resistance index; LVOT- left ventricular outflow tract diameter; LVESD - left ventricular end systolic diameter; LVEDD - left ventricular end diastolic diameter; FS - fraction shortening; CVP - central venous pressure; TEI index - myocardial performance index; CRT - capillary refill time.

Because all the cases included in this study had fluid-resistant septic shock, all required inotropic support for management, and many (62.2%) required more than two inotropic agents.

- Adrenaline was the starting inotrope in 11 (24%) cases; 6 of 11 had low cardiac index (< 3.3L/min/m^2^), and 5 had normal cardiac index (3.3 - 6L/m/m^2^).
- Noradrenaline was used as an add-on vasopressor in 4 (9%) cases (these 4 cases were on adrenaline or dobutamine before adding noradrenaline).
- Dobutamine was the starting inotrope in 32 cases (71%); 20 of 32 cases had normal cardiac index (3.3 - 5.5L/min/m^2^) but with high normal or high SVRI (≥ 1,600dyn-sec/cm^5^/m^2^), and 12 cases had a low cardiac index (< 3.3L/min/m^2^) with high normal or high SVRI.
- Dobutamine was used as an add-on inotrope (inodilator) in 8 cases (these cases were on adrenaline or dopamine before adding dobutamine).
- Milrinone was used as an add-on inotrope (inodilator) in 6 (13%) cases; it is used as an alternative to dobutamine.
- Dopamine was used as the starting inotrope in 2 patients only and as add-on inotrope in 1 patient after dobutamine.
- Nitroglycerin drip was proven to have a good rule in resuscitation of septic shock (cold type) because it was given in 14 (31%) cases with normal or low cardiac index and a high SVRI; it helped in titrated decreases in the SVRI with improvement of the cardiac index (Figure 3).
  **Figure 3:**
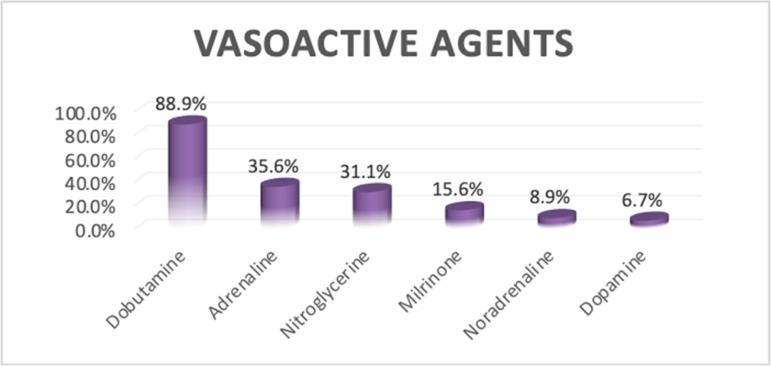
Bar chart describing the different vasoactive agents used in the study.

Nonsurvivors had a higher cardiac output and lower SVRI (they had warm septic shock type on admission). We found that the nonsurvivors had higher inotropes and vasopressor scores at T6 hours (Table 6).

**Table 6:** Comparing cardiac output, systemic vascular resistance index and treatment score between the survivor and nonsurvivor groups

	Mortality (long term)		
	Nonsurvivors (n = 12)	Survivors (n = 33)	p value
CO T0 (mL/min)	1900 ± 741	1564 ± 931	0.021
SVRI T0 (dyn-sec/cm5/m2)	1158 ± 396	1623 ± 550	0.01
Inotropic score T6	22.9 ± 12.5	14.2 ± 6.9	0.012
Vasopressor score T6	26.7 ± 14.8	14.6 ± 6.9	0.008

COP - cardiac output; T0 - time of pediatric intensive care unit admission before inotropic support; SVRI - systemic vascular resistance index; T6 - six hours after inotropic support. Results expressed as mean ± standard deviation.

## DISCUSSION

Shock is considered the main leading cause of morbidity and mortality in the PICU. Septic shock is a serious condition with high mortality rates ranging from 18% to 50%. To improve outcomes, it is important to upgrade hemodynamic monitoring parameters, not just the traditional clinical signs to guide management.^(11)^

Bedside echocardiographic examination was performed to assess the hemodynamic parameters for patients with fluid refractory septic shock. The heart rate, CRT, and base deficit were the clinical and biochemical parameters taken as endpoints of resuscitation. Titration of inotropes and dilators until therapeutic-end points was preceded by measuring the hemodynamic parameters via echocardiographic assessment at fixed time intervals.

The main findings of this study were that VTI, SVI, and cardiac index were compromised at the time of PICU admission (T0) and progressively improved, matching clinical therapeutic target achievement, inotrope and dilator modulation. Fractional shortening was within the normal range and remained there, with no remarkable changes throughout the follow-up period. Furthermore, the Tei index became prolonged (indicating impaired global myocardial performance) at T6 hours and then remained almost unchanged afterward.

These findings were consistent with those of Basu et al., who measured impaired myocardial performance by two-dimensional speckled tracking imaging in patients with septic shock that had not been recognized by conventional echocardiography measuring FS and ejection fraction. They stated that these standard parameters were load-dependent and often revealed cardiac dysfunction only after clinical deterioration had occurred.^(17)^

This impairment of MPI detected in our study can be explained by the fact that global myocardial dysfunction can occur in septic shock and goal-directed therapy helps in preventing worsening of myocardial performance. This is consistent with an adult study done by Nizamuddin et al., which concluded that MPI used to assess subtle changes in myocardial function not detected by routine hemodynamic monitoring, strongly correlated with mortality among patients with severe sepsis and septic shock; changes in MPI may be a prognostic tool in patients with septic shock and may raise the possibility that myocardial dysfunction plays a mechanistic role in sepsis-related mortality.^(18)^

Optimization of cardiac output and SVRI was associated with improved outcomes in fluid-refractory septic shock in children. Similar observations have been made in adults as well, using goal-directed therapy.^(19)^

In this study, the significant increase of the cardiac index was observed by the end of resuscitation of septic shock, consistent with findings of other studies that considered cardiac index to be the standard parameter in resuscitation of septic shock, because attainment of the therapeutic goal of a cardiac index between 3.3 and 6.0L/min/m2 may result in improved survival in patients with septic shock.^(1,2)^ The cardiac index was found to be negatively correlated with SVRI because excessively high SVRI has detrimental effects on the cardiac index; this is consistent with the results of the study done by Deep et al., which found a strong negative correlation between cardiac index and SVRI.^(20)^

In this study, we also observed that SVRI was significantly decreased after 6 hours of resuscitation in comparison to Time 0, and then showed mild rebound elevation at T24 hours with no significant difference. This may be explained by sympathomimetic withdrawal lagging behind cardiac index optimization despite the use of dilators such as nitroglycerine and milrinone. The theory of ischemia/reperfusion should be considered, because the bioavailability of nitric oxide, an important mediator of vasodilation, is profoundly decreased during the reperfusion period, resulting in impaired vasodilation of arterioles. Release of inflammatory mediators and increased expression of adhesion molecules initiate inflammatory and coagulation cascades that culminate in the occlusion of capillaries, known as the "no-reflow" phenomenon.^(19)^ Such a rebound increase in resistance can lead to a decrease in cardiac output, and vice versa. Furthermore, SVRI was observed to be negatively correlated with VTI. Despite the systemic vascular resistance being critical in the infants' compensatory mechanisms, its persistent or rebound elevation has a poor impact on hemodynamic resuscitation parameters.^(21)^

In our study, echocardiographic assessment was helpful in differentiating the type of septic shock (cold and warm). Cold septic shock predominated in our study (82%), whereas warm septic shock represented 12%; nevertheless, it had the highest case fatality (34.4%), and this is consistent with the results of the study conducted by Deep et al., which concluded that the cold type of septic shock is far more common than the warm type.^(20)^

In essence, this provides a strong argument for quantification and consideration of all three circulatory components when treating septic shock: flow, pressure and resistance.^(19)^

The median resuscitation time of septic shock in survivors was 34 hours and was inversely proportional to the SVI assessed after 24 hours, indicating that the SVI is more sensitive than cardiac index as a surrogate of resuscitation of septic shock. In a similar study conducted on 36 children with fluid refractory septic shock, the resuscitation time was 42 hours.^(20)^

The mortality rate in our study was 27%, which was higher than a similar study conducted on patients of the same age group.^(21)^

In the present study, we found that nonsurvivors had warm septic shock type on admission (a higher cardiac output and lower SVRI), which was the type with highest case fatality (34.4%). Despite cold shock predominating community-acquired septic shock, warm shock carries a higher mortality.^(22)^

Our nonsurvivors had significantly lower SVRI than did survivors with a mean of 1,158 ± 396dyn-sec/cm^5^/m^2^; this value was even lower than the value identified by Lee et al., who stated that SVRI of 1,167dyn-sec/cm^5^/m^2^ was a convenient point to predict mortality (measured by pulmonary thermodilution method).^(11)^^)^ This may be explained by the fact that sepsis causes circulatory failure, secondary to vasodilation (especially caused by endothelial injuries).^(23)^

## CONCLUSION

There was a persistently high systemic vascular resistance index in cold shock patients, influencing the stroke volume index, cardiac index and velocity time integral. The use of small doses of dilators and avoiding excessive vasopressor may help to better improve the hemodynamics of critically ill children.
